# Applying Cold Atmospheric Plasma to Preserve the Postharvest Qualities of Winter Jujube (*Ziziphus jujuba Mill. cv.* Dongzao) During Cold Storage

**DOI:** 10.3389/fnut.2022.934841

**Published:** 2022-07-06

**Authors:** Tao Jin, Chenwei Dai, Yong Xu, Yan Chen, Qinghua Xu, Zhengwei Wu

**Affiliations:** ^1^School of Nuclear Science and Technology, University of Science and Technology of China, Hefei, China; ^2^Joint Laboratory of Plasma Application Technology, Institute of Advanced Technology, University of Science and Technology of China, Hefei, China; ^3^Anhui Academy of Medical Sciences, Hefei, China; ^4^Institute of Advanced Technology, University of Science and Technology of China, Hefei, China; ^5^CAS Key Laboratory of Geospace Environment, University of Science and Technology of China, Hefei, China

**Keywords:** cold atmospheric plasma, winter jujube, antioxidant activity, gene expression, postharvest qualities

## Abstract

Winter jujube (*Ziziphus jujuba Mill. cv.* Dongzao) is a very popular horticultural fruit worldwide, which contains a high number of bioactive compounds. Nevertheless, jujube is perishable by microbial contamination and has a short shelf life under non-controlled conditions. Cold atmospheric plasma (CAP) presents a great potential for food sterilization, maintain postharvest quality, and prolonged storage time. Herein, this study investigated the potential effect of CAP with different exposure times (0, 5, 10, and 20 min) on the physicochemical and biochemical changes in jujube during 15-day storage at 4°C and 90% relative humidity (RH). The results showed that CAP treatment could obviously delay ripening, but displayed no effects on the speed of weight loss and moisture content. Meanwhile, the total native aerobic bacterial count in each jujube group was restrained during whole storage. However, CAP treatment showed a time-dependent manner to improve gene expression (PAL, 4CL, DFR, ANS, LAR, and ANR) related to phenolic biosynthesis. As compared to other groups, 20-min CAP treatment can keep or increase total phenolic content (TPC), maintain antioxidant activity, and reduce oxidative damage. Furthermore, the hydrogen peroxide (H_2_O_2_) and malondialdehyde (MDA) content in jujube during middle storage were visibly reduced by 20-min CAP treatment. All in all, our findings concluded that appropriate CAP exposure time can be a promising candidate for the postharvest preservation of jujube.

## Introduction

Winter jujube (*Ziziphus jujuba* Mill. cv. Dongzao) is a traditional plant of China, which has been cultivated for more than 4,000 years. It is regarded as one of the most popular fruits in the world due to its incomparable nutritive value, susceptive aroma, and delicious taste. Winter jujube contains many bioactive compounds such as polysaccharides, ascorbic acid, phenolic acids, amino acid, flavonoids, and mineral constituents, so that displays antioxidant, anti-inflammatory, antimicrobial, and anticancer abilities (1–3). Compared with dried jujube, fresh jujube fruit has more antioxidants and tastes better. However, after harvesting, jujube is highly susceptible to get contaminated by pathogenic including *Alternaria alternata*, *Botrytis cinerea*, and *Penicillium expansum* (4, 5). On the other hand, numerous uncontrollable elements that include respiration, transpiration, temperature, and relative humidity during postharvest storage and transportation have resulted in many quality losses of jujube and critically reduce farmer’s income. Thus, it is of importance to apply an effective strategy to preserve the postharvest quality of jujube during transportation and storage.

So far, various approaches that include food additives, fungicides, and special gas have been developed to retain nutrition, reduce decay, and prolonged shelf life of jujube as far as possible. Hydroxychloroquine (HCQ) (6), melatonin (MET) (7), natamycin (NATA) (8), 1-methylcyclopropene (1-MCP) (9), β-aminobutyric acid (BABA) (10), oligochitosan (OCH) (11), essential mineral (Fe, Zn, Cu, Mn, and Se) mixture (12), carbon monoxide (CO) (13), nitric oxide (NO) (14), and even their combinations were used to enhance antioxidant nutrients, delay senescence, and suppress pathogenic fungi of jujube during postharvest storage (15–18). Moreover, it also has been reported that low temperature (0°C) could be used to delay ripening rate, improve antioxidant levels, and maintain storage quality of jujube (19). Nevertheless, long-term usage of fungicides and pesticides will lead to a negative effect to produce more drug-resistant pathogens. To some extent, residual chemical fungicides located on the surface or inside of fruit will endanger human health. Therefore, the discovery of alternative, easy controlled, lower energy consumption, fruit safe, and environment friendly approaches to preserve the postharvest quality and delay senescence during transportation and storage of jujube are required.

Plasma, known as a chaos state, contains various excited species including O, O_2_, O_3_, OH, NO, NO_2_, and NO_*x*_ and even electric fields, ultraviolet radiation (UV), free radicals, and high-energy particles (20). Cold atmospheric plasma (CAP) is another type of plasma produced under standard pressure and simple gas environment besides thermal plasma and characterized with lower temperature around 300–1,000 K. Over the recent years, cold plasma has shown unique merits and applications in textiles, automotive, aerospace, packaging materials, water purification, clinical medicine, and food industry among others (21, 22). With the increasing demand for safe, convenient, and tasty food of consumer, numerous alternative approaches to preserve the storage quality of fruit and vegetable are needed. According to the previous literature, many studies have well demonstrated that plasma can effectively exterminate pathogens (23), degrade pesticide residue and mycotoxins (24, 25), inactivate metabolism enzymes (26–28), increase bioactive compound contents, and improve antioxidant activity (29). *Liu et al*. pointed out that CAP treatment can reduce rot rate and inhibit *B. cinerea* increment in mulberry fruit (30). Besides, increasing phenolic compounds and antioxidant ability and longer shelf-life of strawberry treated by surface dielectric barrier discharge (SDBD, 6 kV_*pp*_ and 42 kHz) for 10 min and kumquat (*Citrus japonica*) fruits treated by intermittent corona discharge plasma jet (ICDPJ, 8 kV and 4.0 A) for 120 s were acquired (31, 32). As it well known, the preservation effects of cold plasma on postharvest quality of fruits and vegetables are closely related to the carrier gases (air, oxygen, nitrogen, argon, helium, or a combination thereof), generating sources (dielectric barrier discharges (DBDs), corona discharge (CD), microwave discharges (MWs), atmospheric cold plasma jet (APPJ), etc.), treating ways (direct and indirect), and time (33). Nevertheless, to the best of our knowledge, there is rare information about the effects of cold plasma on jujube, but Bao et al. elucidated the cold plasma treatment for 15 s on each side of jujube slice could effectively enhance drying rate and effective diffusivity at 60 and 70°C. Consequently, dried jujube slices with higher polyphenol content and stronger antioxidant capacity are prepared (34). Therefore, it is of importance to unravel the physicochemical and biochemical effects of CAP pretreatment on fresh jujube during cold storage.

Accordingly, the objective of this study was to apply CAP pretreatment to delay ripening and senescence of jujube. Finally, our work will provide a theoretical support for proposing CAP as an effective storage and preservation strategy for jujube fruit.

## Materials and Methods

### Jujube Processing

Winter jujube was harvested from an orchard located Dali, Shaanxi, China and immediately transported to our laboratory within 3 days. A total of 700 jujubes (weight: 12–16 g) of uniform size, color, and without visual defects were collected and randomly allocated into four groups of 175 fruit each.

These jujubes were first disinfected with 200 μl L^–1^ sodium hypochlorite for 2 min and then washed with sterilized distilled water and air-dried prior to use. Afterward, the jujube was treated with the following conditions: (1) for CAP treatment, the jujubes were irradiated with a DBD plasma device (10 mm × 15 mm) at 12 cm distance for 5, 10, and 20 min (labeled as CAP5, CAP10, and CAP 20, respectively) (Supplementary Figure 1). (2) for the control group, jujubes without any treatment were coded as control. After treatment, all jujubes were stored at 4°C and 90 % RH for 15 days. Each sampling experiment was carried out at 5-day intervals. For each group, 10 fruits were used for the measurement of weight loss, and 5 fruits for appearance changes during whole storage. Besides, every 5 fruits were used for evaluating the moisture content and 5 fruits for detecting the total native aerobic bacterial counts. Furthermore, 30 fruits in each group were randomly collected every 5 days, cut into slice, frozen by liquid nitrogen, pulverized, and then stored at –80°C for the assessment of quality parameters including TPC, antioxidant activities, lipid oxidation degree, and phenylpropane pathway-related gene expression.

### Fruit Quality Assessment

#### Visual Appearance

The visual appearance images of jujubes at days 0, 5, 10, and 15 were obtained by a mobile phone in a darkroom with a single light source. After pictured, the jujubes were put back into the incubator and keep stored for the next record till the end of the experiment.

#### Weight Loss

The weight loss of jujubes during 15-day storage was carried out by weight method, using an electronic balance (0.0001 g). The results were calculated by the following equation:


(1) $$\mathrm{Weight}\mathrm{loss}\left(\%\right)=\frac{M_{0}-M_{t}}{M_{0}}\times 100$$

where *M*_0_ represents the initial weight of jujubes at day 0, and *M*_*t*_ means the weight value of jujubes at days 5, 10, and 15, respectively (*t* = 5, 10, 15).

#### Moisture Content

The moisture content of jujubes in each group at every sampling day was determined according to the Chinese standard named GB 5009.3-2016, with some adjustments. A total of 5 jujubes were cut into small slices and put into five glass petri dishes, respectively, followed hot air-dried at 105 ± 5°C for 2 h. Subsequently, the first-dried sample was cooled to ambient temperature and dried again till the final weight was 50 mg lower than the last. The moisture content of jujubes was calculated by the equation below:


(2) $$\mathrm{Moisture}\mathrm{content}\left(\%\right)=\frac{M_{0}-M_{f}}{M_{0}}\times 100$$

where *M*_0_ and *M*_*f*_ symbolize the weight of jujubes before and after hot air-drying treatment, respectively.

#### Total Aerobic Bacterial (TAB) Count Determination

The total native aerobic bacterial count was determined by the National Food Safety Standards of China (GB 4789.2-2016), with slight modifications. A total of 5 jujubes were separately cut into slices, diluted with desterilized normal saline (0.9 % NaCl) at a ratio of 1:10, and tapped for 2 min to prepare the original TAB solution. About 100 μl of above solution was spread on the plate count agar (PCA) medium, cultured at 37 ± 1°C for 48 h. The plates with the number of colonies between 30 and 300 CFU were recorded. Each value was obtained two times by technical replications, and the result was averaged by three values. The results were expressed as log_10_ CFU g^–1^.

#### Antioxidant Activity Determination

The antioxidant activity of jujubes was investigated by 2,2-diphenyl-1-picrylhydrazyl (DPPH) and 2,2′-azinobis-(3-ethylbenzthiazoline-6-sulphonate) (ABTS) methods proposed by the previous study, with some simplified changes (35). About 0.1 g freeze-dried jujube powder was added to a 2-ml tube and immersed with 1 ml of 60% ethanol reagent. Then, the mixture was put into a 60°C water bath for 1 h and around 700 ul extract was obtained. Subsequently, 400 μl extract was mixed with 5.6 ml of 200 μm DPPH solution for 30 min in a darkroom. The absorbance was measured at 515 nm, and the final DPPH scavenging rate was calculated by the equation below:


(3) $$DPPH\mathrm{scavenging}\mathrm{rate}\left(\%\right)=\frac{{\text{A}}_{0}-{\text{A}}_{s}}{{\text{A}}_{0}}\times 100$$

where A_0_ and A_*s*_ represent the absorbance values of the mixture prepared by 400 μl ethanol or 400 μl jujube extract mixed with 5.6 ml DPPH solution, respectively.

Besides, 10 μl jujube extract was reacted with 90 μl ABTS solution for 20 min. Then, the absorbance was read at 732 nm, and the ABTS scavenging rate was calculated followed by the equation below:


(4) $$ABTS\mathrm{scavenging}\mathrm{rate}\left(\%\right)=\frac{{\text{A}}_{0}-{\text{A}}_{t}}{{\text{A}}_{0}}\times 100$$

where A_0_ and A_*t*_ represent the absorbance values of the mixture prepared by 10 μl ethanol or 10 μl jujube extract mixed with 90 μl ABTS solution, respectively.

#### Total Phenolic Content (TPC)

The TPC of jujubes was determined using the Folin–Ciocalteu colorimetric method according to Dzimitrowicz et al., with some modifications (36). About 0.1 g of freeze-dried jujube powder was blended with 1.6 ml of 60% ethanol solution, ultrasonic extracted at 40°C for 2 h, and centrifuged at 5,600 × *g* for 15 min to obtain the TPC extract. Subsequently, 200 μl of extract was brought into a 10-ml tube, and 2 ml of 0.2 M Folin–Ciocalteu reagent was added and vortexed thoroughly. After 5 min, 2 ml of 7.5% (w/v) sodium carbonate solution and 2.8 ml of deionized water were further added and mixed vigorously and put in a darkroom for 30 min. Finally, the absorbance was measured at 765 nm in a microplate reader (CLARIO star, Munich, Germany) with gallic acid used as the standard for calibration curve. The TPC was expressed as g gallic acid equivalence per kg of dry weight (g GAE kg^–1^ DW).

#### Real-Time Quantitative PCR (RT-qPCR)

In this experiment, the major genes involved in phenylpropanoid pathway including PAL, 4CL, DFR, ANS, LAR, and ANR were selected according to Wang et al. (37). Total RNAs from jujubes on days 0, 5, 10, and 15 were extracted with the cetyltrimethylammonium bromide (CTAB) method. RT-qPCR experiment was conducted followed by the manufacturer’s instruction. The results were calculated using 2^–ΔΔ*Ct*^ against the original values of the sample at day 0 as relative expression levels. Finally, the genes and primer sequences designed in the present studies are listed in Table 1, and the actin gene was used as the reference gene. Each sample was carried out for three technical replications, and the results were averaged by three samples.

**TABLE 1:** Gene sequences used in real-time quantitative PCR (RT-qPCR) procedures.

Genes	Primer sequences (5′→3′)
Actin	F: TGGATGATTCTGGCAAAG R: GTAATGGCGGTCAAAGTG
Phenylalanine ammonia-lyase (PAL)	F: AGTGAATGGCACTGCTGTTG R: GACCAGGATGGTGCTTCAAT
4-coumaroyl-CoA synthase (4CL)	F: GACAGACCCTGCTTGATCGT R: GTGGAGACGGCTCCGATTAT
Dihydroflavonol 4-reductase (DFR)	F: GGCTGCCAATAATAGTTGTG R: CTCGACCTTTGTTCATTGCT
Anthocyanidin reductase (ANS)	F: TCTGGATGTTTATGAAGGGA R: CCGATAAATTCCCACTGAGT
Leucoanthocyanidin reductase (LAR)	F: TTATCATGACAACACCCACC R: AGCTCGTTCATGCTCAGTAG
Anthocyanidin synthase (ANR)	F: ACAGTGATCGAATGGGCTCA R: CGCTTGCAAACCCACTGTAA

#### Determination of Hydrogen Peroxide (H_2_O_2_) and Malondialdehyde (MDA) Content

The H_2_O_2_ content was carried out based on titanium sulfate colorimetric method, with slight modifications (38). First, 0.1 g of freeze-dried jujube powder was decolored using 2 ml pre-cooled acetone, accompanied by grinding for 1 min. The mixture (∼2 ml) was collected, the residual jujube powder in mortar was washed by another 3 ml acetone, and 5 ml of jujube-acetone mixture was obtained. After centrifugation at 5,600 × *g* for 15 min, 1 ml of supernatant was added to a 2-ml tube, and 100 μl of 5 % titanous sulfate regnant and 200 μl of ammonium hydroxide were successively added. After reaction for 5 min and centrifuged at 5,600 × *g* for 10 min, the precipitate was preserved and used for next procedure. Finally, the precipitate was washed by acetone for two times and 1.5 ml of 2 M vitriol solution was added. The absorbance value of 150 μl reaction solution was obtained at 415 nm. The standard curve was built using different concentrations of H_2_O_2_ solution by the same processing. The results were expressed as mmol kg^–1^ dry weight (mmol kg^–1^ DW).

The MDA content of jujubes was determined based on the thiobarbituric acid (TBA) method, with some adjustments (39). The absorbance of the supernatant was read at 450, 532, and 600 nm. The MDA concentration was calculated by the following equations:


(5) $$c\,\left(\mu \,m\,o\,l\,L^{-1}\right)=6.45\times \left(A_{532}-A_{600}\right)-0.56\times A_{450}$$

(6) $$\mathrm{MDA}\,\mathrm{concentration}\,\left(\mu \,\mathrm{mol}\,{\mathrm{kg}}^{-1}\,\mathrm{dry}\,\mathrm{weight}\right)=\frac{c\times {\text{V}}_{r}\times {\text{V}}_{e}}{{\text{V}}_{d}\times m\times 1000}$$

where c displays the MDA content of reaction solution, μmol L^–1^; A_450_, A_532_, and A_600_ represent the absorbance values of reaction solution at 450, 532, and 600 nm, respectively; V_*e*_, V_*r*_, and V_*d*_ mean the total volume of sample extract, the volume of sample extract taken for chromogenic reaction, and the volume used for absorbance detection, respectively, ml; m symbols the dry weight of jujube used for extraction, g.

### Statistical Analysis

All data were collected, recorded, and calculated by Excel 2016. Every figure was plotted using Origin 8.5, 2019b version. Moreover, one-way analysis of variance (ANOVA) tools including least-significant difference (LSD) and Waller-Duncan were used to compare and label statistically differences between groups. The results were recorded as mean ± SD (standard deviation) and *p* < 0.05 represented the significant differences.

## Results

### Surface Morphology

**FIGURE 1:**
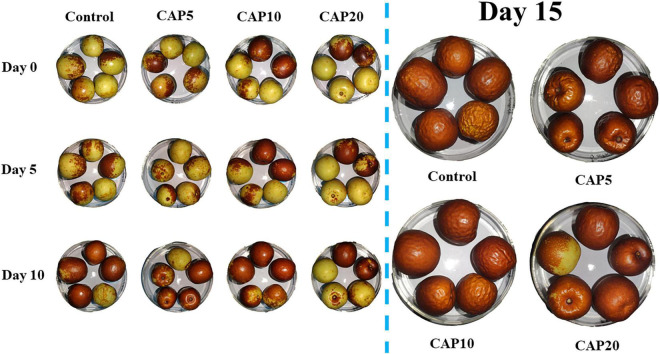
Visual appearance of jujubes in CAP5, CAP10, CAP20, and control during 15-day storage at 4°C and 90% relative humidity (RH).

Figure 1 shows the visual images of jujube during 15-day postharvest storage, which can directly reflect the ripening, senescence, surface morphology, and color change on jujube. At day 10, the jujubes in control, CAP5, and CAP10 are gradually turning red and epidermal tissue is getting dehydrated and shrunk. However, the jujube in CAP20 still displayed a yellow and glossy surface. At the last sampling day, all fruits in control, CAP5, and CAP10 were red, and numerous trenches formed on the surface. Nevertheless, there still were some immature jujubes with half yellow area and highly acceptable appearance in CAP20.

### Weight Loss and Moisture Content

**FIGURE 2:**
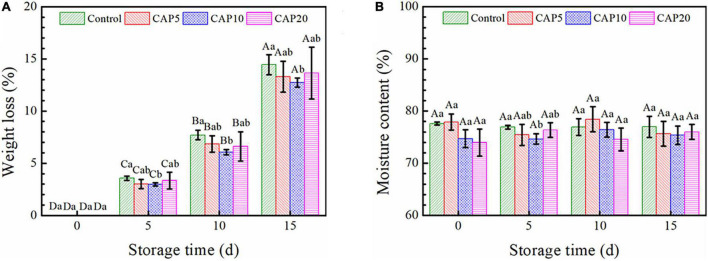
The weight loss **(A)** and moisture content **(B)** of jujubes in CAP5, CAP10, CAP20, and control during 15-day storage at 4°C and 90 % relative humidity (RH). Different small letters indicate significant differences among different groups at the same storage time (*p* < 0.05), whereas different capital letters represent significant differences among different storage times within the same group (*p* < 0.05).

Figure 2 shows the weight loss and moisture content of fresh jujube pretreated or untreated by CAP during 15-day cold storage. As shown in Figure 2A, the weight loss of jujube in every group was gradually increased from days 5 to 15. The weight loss of jujube in CAP5 and CAP20 was slightly decreased as compared to that in control. However, an obviously diminution on weight loss was observed in CAP10 during the whole storage (*p* < 0.05).

As can be seen in Figure 2B, from days 5 to 15, the moisture content of jujube with or without CAP treatment presented no changes as compared to day 0. On the other hand, CAP treatment for 5, 10, and 20 min displayed no effects on the moisture content of jujube at days 0, 10, and 15, whereas the value was decreased in CAP10 group as compared to that in control at day 5 (*p* < 0.05).

### Total Native Aerobic Bacterial (TAB) Count

**FIGURE 3:**
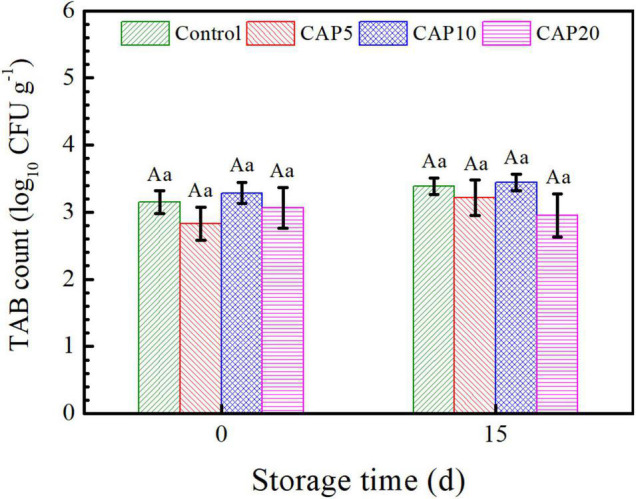
Total native aerobic bacterial (TAB) count of jujubes in CAP5, CAP10, CAP20, and control at days 0 and 15. Small and capital letters show the similar meanings as Figure 2.

As shown in Figure 3, the native TAB count of jujube in CAP5 and CAP20 decreased to 2.83 and 3.07 log_10_ CFU g^–1^, respectively, whereas the number of that in CAP10 was increased to 3.28 log_10_ CFU g^–1^ as compared to control (3.15 log_10_ CFU g^–1^). At the end of 15-day storage, the TAB count of CAP-treated jujube all presented no differences as compared to control. Moreover, with the prolonged storage time, the TAB count of jujube in control and CAP treatment was not changed.

### Antioxidant Ability and Total Phenolic Content (TPC)

The antioxidant ability of fruit and vegetable is strongly associated with antioxidant compounds and usually characterized by the free radical scavenging capacity including DPPH and ABTS. As shown in Table 2, the TPC was gradually decreased with the prolonged storage time. At day 0, the TPC of freeze-dried jujube was reduced by the CAP treatment for 10 and 20 min (*p* < 0.05), whereas there was no difference of that in CAP5 as compared to control. From days 5 to 10, a slight increment of TPC was observed in CAP5, CAP10, and CAP20 as compared to control. However, at the end of 15-day storage, the TPC of jujube in each group was further decreased and still presented no differences with each other.

**TABLE 2:** The antioxidant ability characterized by diphenyl-1-picrylhydrazyl (DPPH)and ABTS scavenging capacity, and total phenolic content (TPC) of jujubes in cold atmospheric plasma 5 (CAP5), CAP10, CAP20, and control during 15-day storage at 4°C and 90% relative humidity (RH).

		Treatment			
	Storage (d)	Control	CAP5	CAP10	CAP20
Total phenolic content (g GAE kg^–1^ DW)	0	23.79 ± 1.08 Aa	23.50 ± 0.06 Aab	21.55 ± 1.12 Ab	21.71 ± 0.24 Ab
	5	19.07 ± 2.99 Ba	21.53 ± 1.26 Ba	20.85 ± 1.75 Aa	20.04 ± 0.09 Ba
	10	16.93 ± 1.65 Ba	18.43 ± 0.84 Ca	18.07 ± 0.80 Ba	18.09 ± 0.64 Ca
	15	15.32 ± 0.08 Ba	15.26 ± 0.43 Da	15.01 ± 0.38 Ca	15.90 ± 0.91 Da
DPPH scavenging capacity (%)	0	80.38 ± 3.13 Aa	82.86 ± 0.81 Aa	81.05 ± 1.89 Aa	83.05 ± 0.36 Aa
	5	80.95 ± 2.52 Aa	82.67 ± 0.36 Aa	83.05 ± 0.13 Aa	83.14 ± 0.47 Aa
	10	82.86 ± 0.23 Aa	83.14 ± 0.00 Aa	81.81 ± 1.72 Aa	83.43 ± 0.23 Aa
	15	83.81 ± 0.13 Aa	82.95 ± 0.49 Ab	82.95 ± 0.36 Ab	83.14 ± 0.40 Ab
ABTS scavenging capacity (%)	0	97.82 ± 0.14 Aa	96.89 ± 1.41 ABa	97.97 ± 0.04 Aa	98.09 ± 0.08 Aa
	5	97.34 ± 0.87 Aa	97.98 ± 0.05 Aa	94.05 ± 3.90 Aa	98.01 ± 0.04 Aa
	10	59.01 ± 10.50 Bb	83.17 ± 9.95 Ba	85.50 ± 8.60 Aa	88.41 ± 10.44 Aa
	15	\	\	\	\

*Data are recorded as mean ± SD. Small and capital letters show the similar meanings as Figure 2.*

For antioxidant activity, the results showed that the DPPH scavenging capacity in CAP-treated groups showed no differences as compared to control from days 0 to 10. However, at day 15, the DPPH scavenging capacity of CAP-treated jujubes was decreased as compared to control (*p* < 0.05). Moreover, the DPPH scavenging capacity of jujube with or without CAP treatment displays no obvious changes during the 15-day storage. Likewise, the same trend was observed in the ABTS scavenging capacity of jujube at day 0 and 5, which displayed no differences of that in CAP-treated groups as compared to control. Differently, at day 10, the ABTS scavenging capacity of jujube in control was sharply decreased to 59.01%, while of that in CAP5, CAP10, and CAP20 still maintained a high level around 85.00% (*p* < 0.05).

### Phenolic Acid Synthesis-Related Gene Expression

**FIGURE 4:**
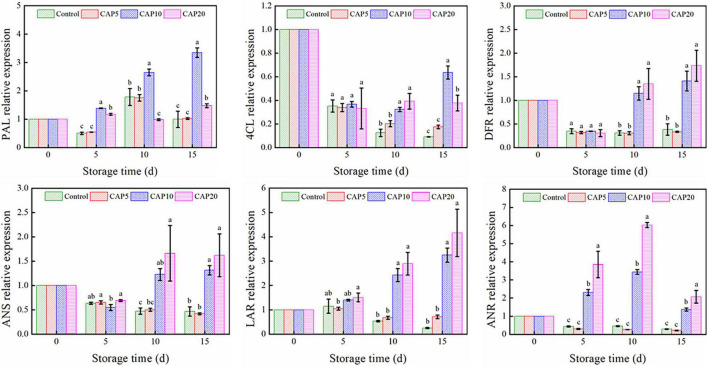
Gene expression of jujubes in CAP5, CAP10, CAP20, and control during 15-day storage at 4°C and 90% relative humidity (RH). Different small letters indicate significant differences among different groups at the same storage time (*p* < 0.05).

To unravel the potential mechanism of CAP treatment on the total phenolic accumulation of jujube during cold storage, the transcriptional expression degrees of 6 genes involved in phenolic biosynthesis were studied. As depicted in Figure 4, CAP treatment for 5 min has no impacts on all 6 genes as compared to control from days 0 to 15. However, 10- and 20-min CAP treatment could improve the PAL, LAR, and ANR expression at days 5 and 10 (*p* < 0.05). Then, at day 15, these genes’ expression declined but still higher than that in control. On the other hand, the expression of 4CL, DFR, and ANS of jujube in control and CAP-treated groups was notably decreased at day 5 as compared to day 0. But from days 10 to 15, 10- and 20-min CAP treatment suppressed the decline rate as compared to control. Furthermore, the expression of DFR and ANS at day 15 was higher than that in control at day 0 (*p* < 0.05).

### H_2_O_2_ and MDA Content

**FIGURE 5:**
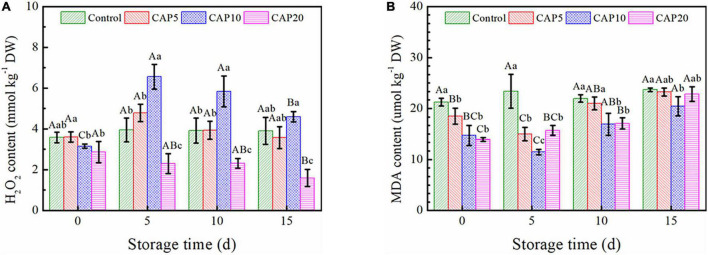
Oxidative stress assessment on H_2_O_2_ **(A)** and MDA **(B)** content of jujubes in CAP5, CAP10, CAP20, and control during 15-day storage at 4°C and 90% relative humidity (RH). Small and capital letters show the same meanings as Figure 2.

As shown in Figure 5A, the H_2_O_2_ content of freeze-dried jujube in CAP10 and CAP20 both decreased at day 0 (*p* < 0.05), while of that in CAP5 was not changed as compared to control. It is noteworthy that there were no concentration changes occurred in control and CAP5 during the whole storage. However, in CAP10 group, the H_2_O_2_ content sharply increased at day 5 and then slowly decreased from days 10 to 15. Furthermore, the H_2_O_2_ content of jujube in CAP20 was lower than that in control and presented a gradually decreasing trend during 15-day storage (*p* < 0.05).

Figure 5B elucidated the MDA accumulation in jujube during 15-day cold storage. At day 0, the MDA content of jujube in CAP-treated groups was reduced and displayed a time-dependent manner. Then, at day 5, the MDA concentration of jujube in control was not changed, while of that in CAP5 and CAP10 was further decreased, and of that in CAP20 start increasing as compared to day 0. As compared to CAP5 and CAP20, the MDA content of jujube in CAP10 was continually increased but still lower than that of control from days 10 to 15 (*p* < 0.05).

## Discussion

In this study, three exposure times of CAP treatment have been used to delay the ripening and senescence of jujube during 15-day storage at 4°C and 90% RH. The results were evident that CAP treatment can reduce the postharvest quality losses and improve oxidative stress response of jujube. As shown in Figure 1, CAP treatment presented a time-dependent manner to postpone turning red and shrinking progress of jujube during 15-day storage. The result was in line with previous study which stated that cherry tomatoes pretreated by CAP activated water still displayed higher acceptable after storage for 14 days (40).

As investigated in Figure 2, CAP treatment can effectively reduce the weight loss of jujube, but this has no concern with moisture content changes. The reason might be due to there were higher content bound water than free water in jujube, and the bound water combined with cell wall polysaccharides and macromolecules was hard to dislodge (41, 42). Besides, the high relative humidity can restrain the water diffusion from peel to air under a stable pressure difference, which already verified by (43–45).

As seen in Figure 3, the result demonstrated that 5-, 10-, and 20-min CAP treatment all have no impacts on TAB count as compared to control. This may be due to 200 μl L^–1^ sodium hypochlorite displayed a brilliant disinfect effect on Jujube and then, the TAB count stood in a safe range (35). Our result was in good agreement with the finding of Won et al., who formulated that *P. italicum* were reduced by microwave-powered cold plasms from 96.6 to 85.0 and 16.0% with an independent input power manner (46). Furthermore, low/ice temperature environment is strictly unfavorable for bacteria growth and proliferation (47).

It is worthwhile to mention that CAP treatment not only can delay decay, reduce weight loss, but also play an important role in antioxidant system. As seen in Table 2, the jujube treated by CAP presented stronger ABTS free radical scavenging capacity and higher TPC than control during middle stage of storage. These differences in antioxidant activity of jujube treated or untreated by CAP were closely depended on the accumulation of antioxidants, which further regulated by the genes related to phenylpropanoid biosynthetic pathway. According to Figure 4, it can be concluded that CAP treatment effectively accelerated the flavonoid and phenylpropanoid biosynthesis in fresh jujube during postharvest storage. In other words, CAP treatment could upregulate key genes’ expression to promote larger phenolic compounds that transform into smaller ones, so that it maintain high-level TPC in jujube. As it is previously reported, CAP treatment could trigger excited stress response to abominable environment through increasing antioxidant compound content in fruits and vegetables, so that defense the oxidative damage (48, 49).

According to the previous studies, H_2_O_2_ is a kind of reactive oxygen species (ROS) produced by stress response in the cells of fruit and vegetable when meet injure, whereas MDA is the main by-product of cell membrane lipid peroxidation in fruits (50). Thus, the H_2_O_2_ and MDA content can reflect the cell membrane permeability, degree of oxidative damage, and fruit aging. Our results showed that short-time CAP treatment could induce seriously the oxidative stress response and increase H_2_O_2_ content, whereas CAP treatment for a longer time would decrease H_2_O_2_ content (Figure 5A). This may be due to longer time exposure can produce more ROS and reactive nitrogen species (RNS), which could effectively lead to serious stress response, so that improve the antioxidant enzyme activities including superoxide dismutase (SOD), catalase (CAT), and ascorbate peroxidase (APX). Rather, short-time treatment could only increase the H_2_O_2_ content and further damage the cell membrane and other tissues of fruits. Besides, the MDA content in our study was first decreased and then increased with a dynamically change trend (Figure 5B). This may be due to CAP treatment not only improved the antioxidant enzymes activity, but also inhibited the lipid peroxidase enzyme expression in jujube during storage. Our results were identical to previous studies, which pointed out that dielectric barrier discharge cold plasma treatment for 10 min can inactivate the litchi peroxidase (POD) to 47.16% (51).

## Conclusion

In this study, a native Chinese winter jujube was used to investigate the potential effects of CAP treatment on the postharvest quality and genes expression. Our results proved that 5-, 10-, and 20-min CAP pretreatment showed no differences on moisture content and total native aerobic bacterial count during 15-day cold storage. However, some meaningful findings were obtained that suitable CAP treatment (10 and 20 min) could reduce oxidative damage and maintain high-level TPC and antioxidant activity through improving key genes’ expression. Taken together, our work demonstrated that CAP treatment can act as a prospective tool to preserve the postharvest qualities of fruits and vegetables. In the later researches, comprehensive effects of CAP on postharvest quality of fruit and vegetable should be further studied including sensory evaluation, color, aroma, texture, polyphenol oxidases, antioxidant enzymes, and cuticle, etc.

## Data Availability Statement

The original contributions presented in this study are included in the article/Supplementary Material, further inquiries can be directed to the corresponding authors.

## Author Contributions

TJ: investigation, methodology, formal analysis, and writing – original draft. CD: methodology and writing – original draft. YX: methodology, validation, and writing – review and editing. YC: data curation, validation, and formal analysis. QX: resources, project administration, and funding acquisition. ZW: conceptualization, resources, writing – review and editing, supervision, and funding acquisition. All authors contribution to the article and approved the submitted version.

## Conflict of Interest

The authors declare that the research was conducted in the absence of any commercial or financial relationships that could be construed as a potential conflict of interest.

## Publisher’s Note

All claims expressed in this article are solely those of the authors and do not necessarily represent those of their affiliated organizations, or those of the publisher, the editors and the reviewers. Any product that may be evaluated in this article, or claim that may be made by its manufacturer, is not guaranteed or endorsed by the publisher.
