# Supplementary material for: Applying Cold Atmospheric Plasma to Preserve the Postharvest Qualities of Winter Jujube (Ziziphus jujuba Mill. cv. Dongzao) During Cold Storage
Source: Front Nutr. 2022 Jul 6;9:934841. doi: 10.3389/fnut.2022.934841 (PMC9298523; doi:10.3389/fnut.2022.934841)
Supplement: Supplementary file 1 [file Image_1.pdf]

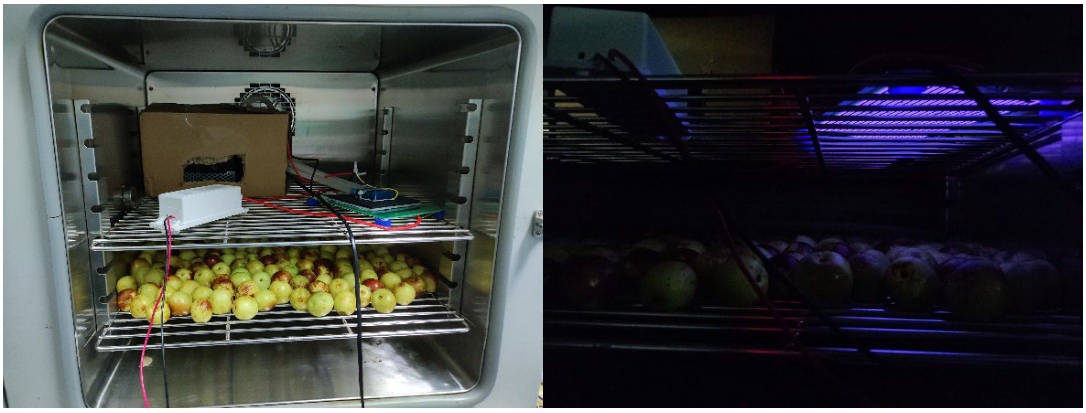

Figure S1. The photos of CAP treatment on jujube. Before treatment (left) and during treatment (right).
